# Translational research in Chagas disease: perspectives in nutritional therapy emerging from selenium supplementation studies as a complementary treatment

**DOI:** 10.1590/0074-02760220001

**Published:** 2022-03-21

**Authors:** Tania C de Araujo-Jorge, Roberto R Ferreira

**Affiliations:** 1 Fundação Oswaldo Cruz-Fiocruz, Instituto Oswaldo Cruz, Laboratório de Inovação em Terapias, Ensino e Bioprodutos, Rio de Janeiro, RJ, Brasil

**Keywords:** trace elements, neglected tropical diseases, myocardiopathy, Trypanosoma cruzi, selenium

## Abstract

Translational research (TR) is an interdisciplinary branch of the biomedical field that seeks to connect its three supporting pillars: basic research on the bench, the hospital beds and other health system services, and the delivery of products for the well-being and health of the community. Here, we review the five transition stages of the TR spectrum, registering the lessons learned during > 20 years leading to the first clinical trial designed and performed in Brazil for testing a complementary treatment for Chagas disease (CD): the selenium trial (STCC). Lessons learned were: (1) to consider all the TR spectrum since the beginning of the project; (2) to start simultaneously animal studies and translation to humans; (3) to ensure a harmonious interaction between clinical and basic research teams; (4) to include MSc and PhD students only in pre-clinical and basic studies (TR0) or vertical clinical studies using retrospective samples and data (TR1); (5) to identify potential suppliers in the national commercial market for a future final treatment since the pre-clinical stage; (6) to keep an international network of experts as permanent advisers on the project. In the whole process, some perspectives were created: a complementary clinical trial for the opened questions and the construction of a Brazilian clinical CD platform.

Chagas disease (CD) is a neglected tropical disease,^1^ distributed over 21 countries, affecting 6-7 million people with 12,000 deaths/year, and considered as a global burden due to migration from Latin America to other continents including Europe, North America, and Asia. Chronic Chagas disease Cardiomyopathy (CCC) is the most relevant infectious heart condition in Latin America. However, translation of knowledge into clinical practices is far from reality. Trypanocide treatment is recommended to prevent or reduce CD progression in both the acute and chronic phases using benznidazole or nifurtimox,^1^ first generation drugs dated from the seventies.

Translational research (TR) is an interdisciplinary branch of the biomedical field that seeks to connect its three supporting pillars: basic research on the bench, the hospital beds and other health system services, and the delivery of products for the well-being and health of the community.^2^ TR is the process of applying laboratory research to human studies and enhancing the adoption of evidence-based practices in real-world settings to reach broad populations.^3^ The main objective of TR is to combine disciplines, resources, knowledge, specialists, and techniques within these three pillars to promote improvements in prevention, diagnosis and therapy. The first studies dated from the 1990s and account now (Sep/2021) for more than 50 thousand records in PubMed. However, TR publications in Tropical Medicine started a decade later and account only to 0.4% of these records. For CD, we only identified 17 records in PubMed, starting in 2004. TR provides the data underlying evidence-based clinical practice and population-based health promotion efforts, including in nutrition and dietetics area.^3^ The time lag estimated to translate research discoveries into day-to-day practices varies from 17 to 24 years^3^ and in neglected infectious diseases this odyssey may attain several decades.

For almost 30 years we have been interested in the role of selenium (Se) in CD pathology, due to two main reasons: malnutrition as consequence of poverty, an important determinant of neglected tropical diseases,^4^ and Se as a relevant micronutrient to cardiovascular health.^5^ From the most relevant trace elements for heath - iron, zinc, and Se - we focused on the last one due to its involvement in some endemic cardiopathies.^5^ However, dietary macro-elements as protein, carbohydrates and lipids are also under study as determinants for CD. A literature review^6^ reinforced “the relevance of nutritional studies in public health for better understanding the aspects involved in the risk and prognosis of malaria, schistosomiasis, visceral leishmaniasis and Chagas’ Disease”. The authors stressed that there were much more experimental studies than population-based studies and “although the first are essential for helping to understand the pathophysiological mechanisms underlying the association between nutritional deficits and those diseases, well designed population-based studies are fundamental for the translation of scientific research into effective actions for controlling neglected diseases”.

CCC is a complex disease with multiple determinants, but its pathogenesis is still under study.^7^ After 1-3 months and a benign acute phase, CD follows a silent and asymptomatic indeterminate phase in immunocompetent hosts and remain mostly in this stage for the lifelong of the affected person. However, in about 25-30% of the seropositive cases, after one or more decades, still unknown triggers lead to a slow progression in severity. CD then evolves from a simple chronic infection to an important organic multi-system disease in which two major clinical forms may occur: a digestive and/or a cardiac form.^7^

Our journey in the translation of CD basic research in mouse models^8^ ^,^ ^9^ into a clinical study^10^ and a clinical trial^11^ earned us many lessons and perspectives about TR. Selenium Treatment in Chagasic Cardiopathy - STCC - is the acronym of our first Se clinical trial.^11^

Lessons learned

*Lesson 1: To consider all the TR spectrum since the beginning of the project* - For any study intending to future translation into clinical practice, it is important to consider all the TR spectrum (Figure) and phases since the first beginning of project conception and elaboration. Basic research (TR0), the initial step for discoveries, includes preclinical and animal studies, aimed to define mechanisms, targets and, relevant molecules. However, animal studies may not inform directly some effects in humans and then a translation to humans (TR1) should be considered as soon as possible. Normal levels and pharmacodynamics of biomarkers in healthy persons may not be the same in patients with a specific disease or infection condition, and a translation to target patients and age range should also be considered in phase 2 and 3 clinical trials (TR2). Then both clinical outcomes and phase 4 clinical trials must be considered if the proposal is to deliver recommended care to the right patients, timely and evidence-based, to translate academic and clinical results into biomedical practices (TR3). Finally, to reach true benefit to society doing translation to community (TR4) it is necessary to perform research at a population level, confirming outcomes at this point. In the five transition stages of the TR spectrum, feed-back movements, adjustments, and course corrections should also be considered, as shown by arrows at left in the Figure. Besides, regulatory aspects should be taken into account in all the TR transition stages. In Se studies on CD, our basic approaches were approved by ethical and scientific committees regarding laboratory animal science, but we only submitted the project to the National Brazilian Regulatory Agency (ANVISA) in the stage of clinical trial brewing, and this added a lag on the time course of the TR spectrum.

**Figure:**
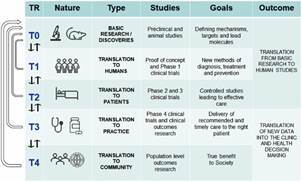
Five transition stages (T0-T4) of the translational research spectrum, adapted from https://tri.uams.edu/about-tri/what-is-translational-research.

*Lesson 2: To start simultaneously animal studies and translation to humans* - With the first question concerning Se deficiency relation to heart inflammation and arrhythmogenesis in CCC we immediately started the two arms of the Se project: (i) pre-clinical experiments in mice, by depleting or supplementing Se in animal diet and following parasitological and inflammatory parameters,^8^ and (ii) vertical studies in CD patients to associate plasma Se levels to the clinical forms.^10^ These first results in humans pushed hard our further studies in mice, thus allowing us to propose mechanisms of action and therapeutic strategies^12^ that have been reinforced by others^13^ and, also, by the results obtained in our clinical trial.^11^ This strategy of starting simultaneously experimental preclinical and clinical human studies was successful, and the two scientific groups interacted since the beginning of the project, leading to lesson 3.

*Lesson 3: To ensure a harmonious interaction between clinical and basic research teams* - Se studies showed us that a continuous monitoring of the specific literature is extremely important for successful TR, and this was possible due to the permanent scientific interaction between the basic science group at the Oswaldo Cruz Institute and the cardiology clinical group at the Evandro Chagas Institute - National Institute of Infectious Diseases, both at Oswaldo Cruz Foundation. Even when changes occurred in both groups, especially in PhD students’ composition, the main scientists remain attached to the project, sustaining its update and facing the difficulties and decisions that were needed at many moments. The well-balanced interaction between clinical and basic research groups is essential to allow TR to proceed from initial ideas until a first human trial.

*Lesson 4: To include MSc and PhD students only in pre-clinical and basic studies (TR0) or vertical clinical studies using retrospective samples and data (TR1)* - During the phase 2 and 3 clinical trials (TR2) it is essential to include in the research team only mature professionals that are economically stable to avoid staff shortages during the trial. We learned this lesson when we proposed parts of the STCC as 4-years PhD projects and faced so many barriers and difficulties in feasibility that two students needed to change their original PhD projects. The conditions to perform basic research are easier to attain than to perform clinical research in humans. Good ideas plus good quality animal facilities plus adequate financial support, science ethics and research integrity are generally sufficient to obtain good basic research in a 2-to-4-year period. However, the timeline for TR involves other regulatory issues with specific training in good clinical research practices plus a higher budget to allow certificated laboratory testing (not in house tests) and patient support that, in our experience, were harder to sustain in long term.

*Lesson 5: To identify potential suppliers in the national commercial market for a future final treatment since the pre-clinical stage* - It was a hard barrier in STCC.^11^ Our first supplier for Se batches to be used in the clinical trial failed to sustain the original good manufacturing practices manufactured supply, and a shortage occurred leading to an unexpected lag period in the project, until a new supplier was found, and the necessary agreements were signed. Thus, we learned this lesson in the worst way, under the threat of trial cancellation.

*Lesson 6: To keep an international network of experts as permanent advisers on the project* - In 2004, our first experimental and clinical publications attracted the attention of the international scientific community and, we were invited to participate biannually of the meetings of the International Society for Trace Elements Research in Humans (ISTERH). This allowed an immersion in an expert community leading to a deep interaction since then and helped us to overcome the numerous doubts that arise in such a long study. In our case, it helped us a lot in questions related to the clinical: use of different Se formulations and presentations, about the physiopathology of cardiovascular diseases and the role of Se, regarding the clinical management of patients during treatment and/or supplementation with Se and management of CD patients evolving with cardiopathy.

Perspectives

The results of STCC clinical trial showed that a potential beneficial influence of Se was observed only in the subgroup of patients at B2 stage,^11^ and, in one-year of follow-up, Se treatment did not improve significantly the overall left ventricular ejection fraction in patients taking 100 mcg/day of Se as compared to the placebo group. We concluded that “complementary studies are necessary to explore diverse Se dose and/or associations in different CCC stages (B2 and C), as well as in A and B1 stages with longer follow-up”. In brief, “patients in stage A present typical CD ECG changes but not ECHO abnormalities; stage B patients present wall motion abnormalities and according to LVEF can be divided in B1, LVEF ≥ 45%, and B2 < without heart failure (HF); stage C patients have LVEF < 45% plus HF symptoms and, in case of end-stage HF, the patient is reclassified as stage D”.^11^

Therefore, the perspectives are clear: (1) to carry out the cardiological follow-up of the study participants for another 4 to 10 years; (2) to request permission from the Ethics Committee to offer Se treatment to those who took a placebo in STCC; (3) to conclude the study of immunological biomarkers and gene polymorphism for cytokines and proteins involved on Se metabolism in the STCC participants samples; (4) to contact suppliers to enable the new studies with Se, preferably multicenter and international. An important development would be a Brazilian platform for clinical studies on CD in endemic areas in a multicentric network, inspired by the Bolivian example and the Nhepacha network experiences.^14^ In Brazil, we intend to incorporate Chagas Express XXI (CE21) as a cutting-edge social educational technology to help in this achievement,^15^ since we demonstrated its potential as an instrument of field epidemiology. CE21 is part of STCC social legacy and was created as an “imaginary train” with more than 40 ArtScience workshops, games, laboratory activities and conversation circles. CE21 was conceived as a social technology since all processes were co-created by scientists and CD patients and worked with local cross-sector partnerships. CE21 is a potentially useful social technology for health and science education and for active search of chronic cases of disease of asymptomatic CD, contributing to the notification of chronic cases and their inclusion in the lines of care and clinical trials. Furthermore, CE21 could be adapted to understand and cooperate in other potentially epidemic situations, especially related to other neglected diseases, such as Leishmaniasis, Tuberculosis and arboviruses.
